# An Adaptive Information Borrowing Platform Design for Testing Drug Candidates of COVID-19

**DOI:** 10.1155/2022/9293681

**Published:** 2022-04-22

**Authors:** Liwen Su, Jingyi Zhang, Fangrong Yan

**Affiliations:** State Key Laboratory of Natural Medicines, Research Center of Biostatistics and Computational Pharmacy, China Pharmaceutical University, Nanjing, Jiangsu Province, China

## Abstract

**Background:**

There have been thousands of clinical trials for COVID-19 to target effective treatments. However, quite a few of them are traditional randomized controlled trials with low efficiency. Considering the three particularities of pandemic disease: timeliness, repurposing, and case spike, new trial designs need to be developed to accelerate drug discovery.

**Methods:**

We propose an adaptive information borrowing platform design that can sequentially test drug candidates under a unified framework with early efficacy/futility stopping. Power prior is used to borrow information from previous stages and the time trend calibration method deals with the baseline effectiveness drift. Two drug development strategies are applied: the comprehensive screening strategy and the optimal screening strategy. At the same time, we adopt adaptive randomization to set a higher allocation ratio to the experimental arms for ethical considerations, which can help more patients to receive the latest treatments and shorten the trial duration.

**Results:**

Simulation shows that in general, our method has great operating characteristics with type I error controlled and power increased, which can select effective/optimal drugs with a high probability. The early stopping rules can be successfully triggered to stop the trial when drugs are either truly effective or not optimal, and the time trend calibration performs consistently well with regard to different baseline drifts. Compared with the nonborrowing method, borrowing information in the design substantially improves the probability of screening promising drugs and saves the sample size. Sensitivity analysis shows that our design is robust to different design parameters.

**Conclusions:**

Our proposed design achieves the goal of gaining efficiency, saving sample size, meeting ethical requirements, and speeding up the trial process and is suitable and well performed for COVID-19 clinical trials to screen promising treatments or target optimal therapies.

## 1. Background

COVID-19 has affected our lives in an all-round way since its first outbreak in 2019. Subsequent waves of case spikes have swept nearly every country, causing considerable morbidity and mortality as coronavirus, and its variants continue to spread and mutate [1]. According to Johns Hopkins' data, 222.5 million confirmed cases and 4.5 million deaths were reported till Sep 9, 2021 [2]. Approximately, 80% of COVID-19 patients had mild or moderate disease, while 14% of patients experience a severe disease course, with case-fatality rates ranging from 0.3 to 7.2% of all confirmed cases [3–5]. Due to its high transmission with a basic reproduction number (R0) of between 1.4 and 7.23 [6, 7] and substantial effects on disease burden, effective treatments are a major concern to fight against this pandemic.

Thousands of interventional studies have been registered in ClinicalTrials.gov related to COVID-19, and this number is increasing progressively. Up to now, 11 therapies for COVID-19 have gained emergency use authorization by the US Food and Drug Administration (FDA) [8], inspiring the following rapid development for treatments of COVID-19. However, the particularity of pandemic disease inevitably complicates clinical trials, making it different from traditional randomized controlled clinical trials. It is mainly reflected in the following aspects: first is the timeliness [9, 10]. A large number of cases and deaths in a short time poses unprecedented pressure on the conventional drug discovery paradigms. The screening of effective treatment drugs has a strong timeliness, calling for more innovative trial designs. Second is the exploration of new indications of existing drugs rather than De Novo drug design, such as the repurposed lopinavir-ritonavir [11]. In these circumstances, the toxicity profile of drugs has been well studied and efficacy evaluation is of top interest. A quick-start phase II trial can get more attention from researchers. The platform trial recommended by FDA [12] can well adapt to these characteristics. WHO also pointed out that integrating clinical trials of candidate therapeutics as part of the response during infectious disease outbreaks is increasingly recognized as important for screening potential drugs. Three is the transient case peak. Due to the strict control of the epidemic after case spike, the sample size of a trial will soon be greatly challenged after the epidemic remains stable, which brings great difficulties to the process of clinical trials, such as two already terminated trials for evaluating Remdesivir in China [13]. Another lesson that should be learned is that quite a few trials on the antiEbola virus began after the Ebola epidemic has alleviated [14]. Therefore, when designing clinical trials for COVID-19, a timely response is necessary, which can be settled by introducing early stopping criteria and making full use of all available data, including information from other drugs' trials, to improve the efficiency of trials.

There have been several platform designs for COVID-19, including RECOVERY [15], REMAP-CAP [16], ACCORD [17], SOLIDARITY [18], and others. These platform designs are all parallel designs, in which interested drug candidates start recruiting patients at the same time (Saville and Berry [19], Yuan et al. [20], and Tang et al. [21]). Their control arms remain unchanged although effective drugs graduate, resulting in ethical problems of not assigning the latest effective drugs to patients. Moreover, concurrent comparison leads to insufficient use of historical control arm information. In consideration of the rapid outbreak of COVID-19, countless patients urgently need effective drugs, so it may be more important to give patients effective drugs as soon as possible. The sequential design may be more suitable under such circumstances. It compares a candidate drug with a standard of care (SOC) under a unified framework sequentially. If declared efficacious, the candidate drug will be added to the control arm and continued to compare with new drug candidates, making it possible for COVID-19 patients to always receive the latest treatments. The treatment in the current control arm is either consistent with that in the previous control arm or experimental arm, thus information from previous stages can be borrowed. Furthermore, due to the variability of the coronavirus, SOC may rapidly evolve and the epidemic has a strong tendency to shift populations (older to younger and back again) during any study. Thus, the baseline effectiveness of SOC may drift over time. Such time trend calibration must be considered when modelling as well.

Borrowing information could improve the power of the trial and adaptive randomization is combined with it to allow more patients to be enrolled into the experimental arm, making it more efficient. Methods have been recently introduced to borrow information. Pocock et al. [22] considered the difference in model parameters between historical data and current data and regarded this difference as a random variable. Ibrahim and Chen et al. [23–25] proposed the power prior method, in which the prior is constructed by raising the likelihood of historical data to the prespecified power *α*. Chu et al. [26] used a calibrated method to measure the heterogeneity and determine *α* for binary endpoints. A number of improvements to the power prior method have been described in the literature [27–29]. Early stopping is another feature of the proposed design to cater to the timeliness characteristics of COVID-19. With early stopping for efficacy and futility, once there is enough evidence to declare effectiveness, drugs are graduated to the next stage or stopped, saving sample size and accelerating the development.

Due to the high infectivity and relatively low mortality of COVID-19, most trials choose time-to-event endpoints. Because the effect size of drugs is extremely limited in case of low mortality, the use of traditional binary endpoints will lead to an excessive sample size. In addition, using time-to-event endpoints can better reflect changes in disease status and cater to changing epidemic characteristics [30].

Based on the above considerations, in the framework of sequential platform design, we integrate power prior and time trend calibration into the platform design and extend it to time-to-event endpoints, thus proposing a COVID-19 sequential platform design that adaptively borrows information from previous stages based on the heterogeneity between stages. The proposed design allows two strategies: the comprehensive screening strategy that aims to screen all drugs that may be effective and the optimal screening strategy that aims to screen the most effective drug. The outline of this article is as follows: Section 2 is a detailed introduction of the proposed model.  Section 3 has the simulation results and Section 4 is a specific example of the design. We conclude with a brief discussion in Section 5.

## 2. Method

In this study, we propose an adaptive information borrowing platform design, which sequentially enrolls patients to either the experimental arm or control arm and makes decisions after data are available. If one drug shows enough efficiency, it will graduate and be added to the control arm, after which a new drug arm will be open for recruitment. Borrowing information happens between the same treatments.

**Figure 1:**
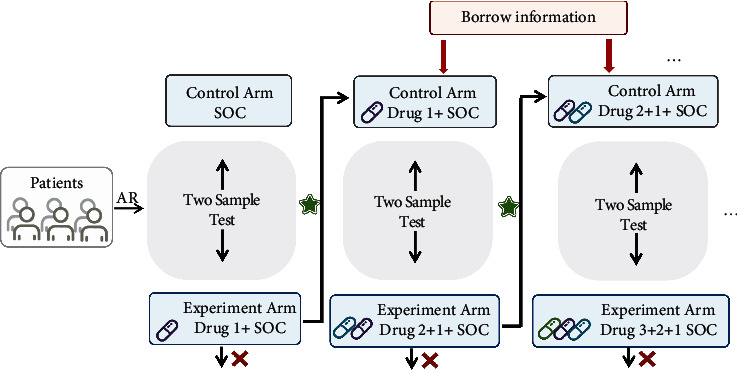
Diagram of platform design for COVID-19.

The overall process of the trial is shown in Figure 1. Considering an exploratory trial with the endpoint being time to clinical remission, for experimental arm A, let *T*_*A*_ denote the time from enrollment to clinical remission. The smaller the remission time, the faster the clinical remission reaches, thus the more effective the drug is. Assuming *T*_*A*_ follows an exponential distribution with hazard *θ*_*A*_

(1) $$\begin{array}{c} T_{A}∼\text{Exp}\left(\theta_{A}\right). \end{array}$$

Let *n*_*A*_ denote the number of patients assigned to the experimental arm A. For patient *i*, let *T*_*i*_^*A*^ denote the observed time and *T*_*i*_ denote the true clinical remission time. *δ*_*i*_ indicates whether to censor, if censoring occurs, *T*_*i*_^*A*^ < *T*_*i*_, *δ*_*i*_=0, if clinical remission occurs, *T*_*i*_^*A*^=*T*_*i*_, *δ*_*i*_=1. Let *S*(*t*)=Pr(*T*_*A*_ > *t*)=exp(−*tθ*_*A*_) represent the survival function. Given data *D*_*A*_={(*T*_*i*_^*A*^, *δ*_*i*_), *i*=1,…, *n*_*A*_} during the interim analysis of the experimental arm A, let *m*_*A*_=∑_*i*=1_^*n*_*A*_^*δ*_*i*_ represent the number of achieving clinical remission and ${\bar{T}}_{A}=\sum_{i=1}^{n_{A}}T_{i}^{A}$ represent the total observation time, we have the likelihood function:

(2) $$\begin{array}{c} L\left(D_{n_{A}}|\theta_{A}\right)=\prod_{i=1}^{n_{A}}f{\left(T_{i}^{A}|\theta_{A}\right)}^{\delta_{i}}S{\left(T_{i}^{A}|\theta_{A}\right)}^{1-\delta_{i}}=\theta_{A}^{m_{A}}\exp\left(-{\bar{T}}_{A}\theta_{A}\right). \end{array}$$

Let *θ*_*A*_ follow gamma distribution *θ*_*A*_ ~ Gamma(*a*, *b*), then *θ*_*A*_ has the posterior distribution:

(3) $$\begin{array}{c} \theta_{A}|D_{A}∼\text{Gamma}\left(a+m_{A},b+{\bar{T}}_{A}\right). \end{array}$$

For control arm B, we assume the time from enrollment to clinical remission *T*_*B*_ also follows an exponential distribution, thus the hazard *θ*_*B*_ has the same distribution as that in the experimental arm A. So, we have the posterior distribution:

(4) $$\begin{array}{c} \theta_{B}|D_{B}∼\text{Gamma}\left(a+m_{B},b+{\bar{T}}_{B}\right), \end{array}$$

where *D*_*B*_, *m*_*B*_, and ${\bar{T}}_{B}$ are defined in a similar way as *D*_*A*_, *m*_*A*_, and ${\bar{T}}_{A}$. Suppose there are *K* interim looks, which occur when the number of enrolled patients reaches *n*_1_,…, *n*_*K*_. Because fewer enrolled patients in the early stage may lead to unreliable estimates, we start the interim analysis until *n*_0_ patients are enrolled and perform an interim analysis for every *n*_*k*_ patients enrolled, up to a maximum sample size of *n*_*K*_. At each interim analysis, if Pr(*θ*_*A*_ > *θ*_*B*_*|D*_*A*_, *D*_*B*_) ≥ *C*_*I*_, the drug in experimental arm A is declared effective and vice versa. After reaching the maximum sample size in each arm, if the risk in experimental arm A is lower than that in control arm B, that is Pr(*θ*_*A*_ > *θ*_*B*_*|D*_*A*_, *D*_*B*_) ≥ *C*_*F*_, the experimental arm A is declared more effective than control arm B. *C*_*I*_ and *C*_*F*_ are obtained by calibration.

### 2.1. Borrow Information

The treatment in the current control arm is either the same as the historical control arm or experimental arm. Specifically, when the drug in the previous stage is effective, we add a “graduated” drug into the control arm, thus the treatment in the current control arm is the same as that in the historical experimental arm. When the drug in the previous stage is ineffective, we remain the treatment in the control arm unchanged, thus the treatment in the current control arm is the same as that in the historical control arm. Therefore, we could use the power prior to borrow information for the current control arm B from previous stages. The power prior method uses the posterior of historical data as the prior of the current parameter. Assuming that the historical data is *D*_*H*_, the initial prior of the parameter *θ* is *π*_0_(*θ*), we have the power prior *π*(*θ*):

(5) $$\begin{array}{c} \pi \left(\theta |D_{H},\alpha \right)\propto {\left[L\left(\theta |D_{H}\right)\right]}^{\alpha }\pi_{0}\left(\theta \right), \end{array}$$

where *α* is the parameter controlling how much to borrow from historical data. The hazard in the current control arm *θ*_*B*_ follows gamma distribution *π*_0_(*θ*_*B*_) ~ Gamma(*a*, *b*). Given historical data *D*_*H*_={(*T*_*i*_^*H*^, *δ*_*i*_), *i*=1,…, *H*}, ${\bar{T}}_{H}=\sum_{i=1}^{n_{H}}T_{i}^{H}$, *θ*_*B*_ has the power prior:

(6) $$\begin{array}{c} P\left(\theta_{B}|D_{H}\right)\propto \theta_{B}^{a-1}\exp\left(-b\theta_{B}\right)\theta_{B}^{\alpha m_{H}}\exp\left(-\alpha \theta_{B}{\bar{T}}_{H}\right)\\ \;\propto \theta_{B}^{a+\alpha m_{H}-1}\exp\left(-\left(b+\alpha {\bar{T}}_{H}\right)\theta_{B}\right)\\ \theta_{B}|D_{H}∼\text{Gamma}\left(a+\alpha m_{H},b+\alpha {\bar{T}}_{H}\right). \end{array}$$

Given the current control arm data *D*_*B*_={(*T*_*i*_^*B*^, *δ*_*i*_), *i*=1, ..., *n*_*B*_}, *θ*_*B*_ has the posterior distribution:

(7) $$\begin{array}{c} \theta_{B}|D_{H},D_{B}∼\text{Gamma}\left(a+\alpha m_{H}+m_{B},b+\alpha {\bar{T}}_{H}+{\bar{T}}_{B}\right). \end{array}$$

Once all the candidate therapies are tested, two drug development strategies are applied:

(1) *Comprehensive Screening Strategy*. This strategy aims to screen all drugs that may be effective, that is, all drugs that satisfy the following rules will be declared effective and enter the next stage:
  (8) $$\begin{array}{c} \mathrm{Pr}\left(\theta_{A}>\theta_{B}|D_{H},D_{A},D_{B}\right)=\int_{0}^{1}G_{B}\left(p|D_{B},D_{H}\right)g_{A}\left(p|D_{A}\right)\text{d}p\\ =\int_{0}^{1}\frac{\gamma \left(a+\alpha m_{H}+m_{B},\left(b+\alpha {\bar{T}}_{H}+{\bar{T}}_{B}\right)p\right)}{\Gamma \left(a+\alpha m_{H}+m_{B}\right)}\frac{{\left(b+{\bar{T}}_{A}\right)}^{a+m_{A}}}{\Gamma \left(a+m_{A}\right)}p^{a+m_{A}-1}\exp\left\{-\left(b+{\bar{T}}_{A}\right)p\right\}\text{d}p, \end{array}$$
where *γ*(·, ·) is the lower incomplete gamma function.
(2) *Optimal Screening Strategy.* This strategy aims to screen the most effective drug, that is, the one with the highest efficacy will be declared optimal and enter the next stage. For example, if drug 1 satisfies rule (8), drug 1 + SOC will replace the old control arm and compare with the follow-up new drugs. At the end of the trial, the last drug that declares effective will be selected as the optimal drug.

### 2.2. Time Trend Calibration

Platform designs that run over a relatively long period may face a baseline effectiveness drift [31], which is reflected in the different hazard ratios between stages. Modelling for such drift in the SOC over time is needed; otherwise, it would result in type I error inflation and power reduction. Here, we add a time trend calibration to measure the drift in different stages, thus *α* becomes data driven rather than prespecified. Specifically, two types of data are available at interim analysis: count data (the number of patients who achieve clinical remission) and survival data (time to clinical remission for each individual). We use chi-square statistic *χ*^2^ to measure the heterogeneity for count data and t-statistic *τ* for survival data. Monitoring one indicator is not enough to reflect all the information, so we calculate *α* by synthesizing information from remission numbers and survival time:

(9) $$\begin{array}{c} \alpha ={\left(\frac{1}{\gamma \left(\chi^{2}+\tau \right)}\right)}^{\rho }, \end{array}$$

where *γ* and *ρ* are the tuning parameters, which are calibrated by simulation to keep type I error in control. Larger *χ*^2^ and *τ* indicate that the information between two stages is heterogeneous, thus *α* will be smaller and we nearly borrow no information and vice versa.

### 2.3. Adaptive Randomization

After posterior inference based on borrowing information, patients are randomized to different arms. Traditionally, we use equal randomization in most cases. However, if a fixed allocation ratio of 0.5 is still used, it is easy to cause an imbalance in the amount of effective information between arms. Therefore, the adaptive randomization is considered to balance information and maximize power, which is achieved by taking the allocation ratio as a function of the effective sample size. According to Hobbs et al. [32], we assume the relationship between sample size and precision is linear, then the effective sample size is approximately the effective sample size of the “borrowed” posterior distribution *n*_*B*_(Prec(*θ|D*_*B*_, *D*_*H*_)/Prec(*θ|D*_*B*_)) minus the sample size of the current control arm *n*_*B*_, which is calculated as follows:

(10) $$\begin{array}{c} \text{ESS}=n_{B}\left(\frac{\text{Prec}\left(\theta |D_{B},D_{H}\right)}{\text{Prec}\left(\theta |D_{B}\right)}-1\right), \end{array}$$

where Prec(*θ*)=[*E*_*θ|D*_{*θ* − *E*_*θ|D*_(*θ*)}^2^]^−1^, denoting the precision of the posterior distribution of *θ*.

At the beginning of the experiment, 1:1 allocation is used. Until a certain amount of information is accumulated, adaptive randomization is performed. *n*_*A*_^*∗*^(*t*) and *n*_*B*_^*∗*^(*t*) are the effective sample sizes of the experimental arm and the control arm during the midterm analysis *t*, and ESS(*t*) represents the estimated effective sample size of the control arm. *R* represents the number of remaining patients to be randomized. The aim is to balance the effective information between two arms. *τ* is the randomized allocation ratio. After adaptive randomized allocation, there is *n*_*A*_^*∗*^(*t*)+*τR*=ESS(*t*)+*n*_*B*_^*∗*^(*t*)+(1 − *τ*)*R*, so *τ* can be solved:

(11) $$\begin{array}{c} \tau \left(t\right)=\frac{1}{2}\left(\frac{\text{ESS}\left(t\right)+n_{B}^{*}\left(t\right)-n_{A}^{*}\left(t\right)}{R}+1\right). \end{array}$$

Because the effective sample size has no upper and lower limits, the range of *τ* defined by the above formula is not limited to [0,1], which does not meet the actual requirements. Therefore, the above formula is changed to posing a limitation to the range [*p*_min_, *p*_max_]:

(12) $$\begin{array}{c} \tau \left(t\right)=\max\left[\min\left\{\frac{1}{2}\left(\frac{\text{ESS}\left(t\right)+n_{B}^{*}\left(t\right)-n_{A}^{*}\left(t\right)}{R}+1\right),p_{\max}\right\},p_{\min}\right]. \end{array}$$

### 2.4. Trial Process

Steps for implementing our proposed design are as follows:

- Step 1: for the first drug, enroll *n*_0_ patients and equally randomized to two arms.
- Step 2: collect data for the first drug and fit model (2), conduct interim analyses, and calculate Pr(*θ*_*A*_ > *θ*_*B*_*|D*_*A*_, *D*_*B*_). If the posterior probability across the cutoff, stop enrollment and declare the first drug effective; otherwise, continue to enroll another *n*_*k*_ patients.
- Step 3: repeat step 2 until reaching the maximum sample size *n*_*K*_. Conduct the final analysis and calculate Pr(*θ*_*A*_ > *θ*_*B*_*|D*_*A*_, *D*_*B*_) to see if the first drug is effective.
- Step 4: for the next drug, enroll *n*_0_ patients. They are still equally randomized to the two arms since fewer enrolled patients in the early stage may lead to unreliable estimates.
- Step 5: collect data for the next drug and fit model (2). At interim look 1, calculate Pr(*θ*_*A*_ > *θ*_*B*_*|D*_*A*_, *D*_*B*_) and start adaptive randomization. At interim look *k*, fit model (2) and calculate Pr(*θ*_*A*_ > *θ*_*B*_*|D*_*A*_, *D*_*B*_). If the previous drug is effective, *D*_*H*_ comes from experimental arm of the previous drug; otherwise, comes from the control arm. If across the cutoff, stop enrollment and declare the next drug effective; otherwise, continue to enroll another *n*_*k*_ patients.
- Step 6: repeat step 5 until reaching maximum sample size *n*_*K*_. Conduct the final analysis and calculate Pr(*θ*_*A*_ > *θ*_*B*_*|D*_*A*_, *D*_*B*_) to see if the next drug is effective.
- Step 7: repeat steps 1–6 until all candidate drugs are tested. Make the final decision on which drugs are effective with the comprehensive screening strategy and which drugs are effective with the optimal screening strategy.

## 3. Simulation

In this section, we run simulations to evaluate the performance of the proposed design. Suppose a platform trial with 5 candidate drugs and 1 SOC. The risk of the control arm is set to *θ*_*B*_=0.2. According to exponential distribution, the average time to clinical remission in the control arm is $\bar{T}=1/\theta =5$ weeks. Risk ratio HR > 1 means that the experimental arm has a greater risk than the control arm, that is, the mean remission time is shorter and the effect is better. Among the 5 drugs in each scenario, bold text indicates effective drugs, and the others are ineffective drugs. When the drug is effective, the probability of rejecting the null hypothesis represents power; while ineffective, the probability of rejecting the null hypothesis is the type I error. We control the type I error to 0.1 through simulation. For the platform design that does not borrow information, the allocation ratio is always 1:1.

**Figure 2:**
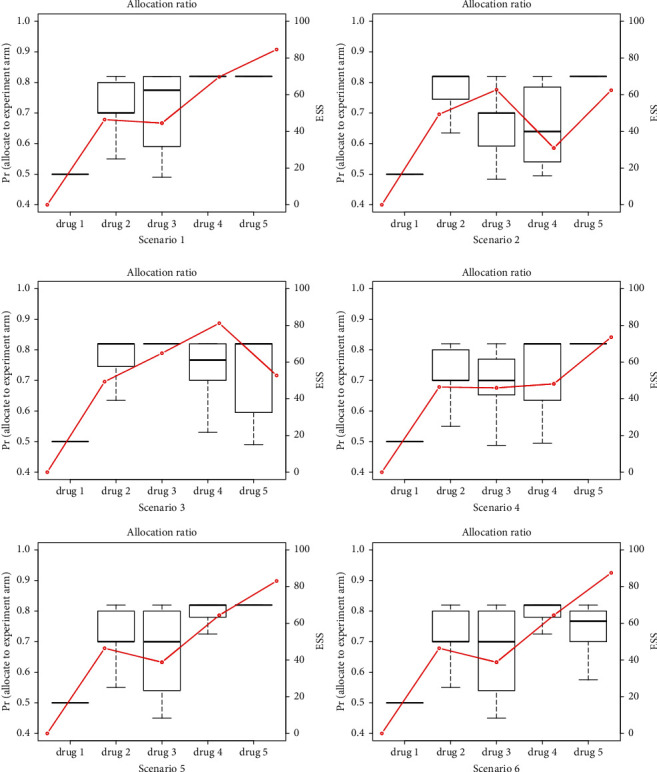
Proportion of patients assigned to the experimental arm and effective sample size.

First, we study scenarios without baseline effectiveness drift by applying the comprehensive screening strategy.  Table 1 lists the simulation results of the COVID-19 platform design using the power prior method with *α* fixed at 0.5. In all 6 scenarios, from the results of the Pr (reject *H*_0_) for the two designs, we can see that the type I error is below 10%. When the drug is truly effective, the proposed design has the power of more than 85% and is higher than that without borrowing information. Pr (early stopping for efficacy) and Pr (early stopping for futility) show the probability of early efficacy/futility stopping. It can be seen that when the drug is truly effective, the probability of effective stopping in most scenarios is more than 65%. When the drug is highly effective, the probability of early stopping can reach more than 95%. While the drug is less effective than the control arm, the probability of futility stopping is about 58%, which allows effective or ineffective drugs to end the trial as soon as possible to speed up the new drug development and save sample size. However, when the efficacy difference of the candidate drug and the control arm is not significant, that is, HR = 1, because we cannot conclude that the drug is effective or ineffective, the trial continues. In terms of sample size, the actual sample size in the scenario with a high early stopping probability is much smaller than the prespecified sample size, in which we can save almost 80–120 patients. As can be seen from *N*_*A*_ and *N*_*B*_, compared with nonborrowing method, the proposed design allocates more patients to the experimental arm. With the trial progressing, the control arm can borrow more information (shown by effective sample size), so the proportion of patients allocated to the experimental arm is also increasing. To further study the impact of the accumulated information on the allocation ratio, we compared the relationship between the proportion of patients assigned to the experimental arm and the effective sample size in each scenario, as shown in Figure 2.

Figure 2 shows how the allocation ratio and accumulated information change with the progress of the trial. Taking scenario 1 as an example, candidate drug 1 has no accumulated information, so the allocation ratio is 0.5. When drug 2 is tested, because the control arm is still SOC, the information of drug 1 could be borrowed, thus more patients are assigned to the experimental arm in drug 2. Drug 2 is declared effective. At this time, when the control arm is drug 2 + SOC, the information of the drug 2 experimental arm in drug 3 can be borrowed, so the amount of information does not change much. From Figure 2, the effective sample size of drug 2 and drug 3 is approximately equal. In drugs 4 and 5, because the information in the control arm accumulated, the effective sample size continued to increase, and the proportion allocated to the experimental arm also increased. However, due to the limitation of the maximum allocation ratio of formula (5), the proportion allocated to the experimental arm was finally constant at around 0.85. From the relationship between the proportion of patients assigned to the experimental arm and the effective sample size above, we can see that the more information accumulated, the greater the effective sample size, thus the higher the proportion of patients assigned to the experimental arm, which meets the ethical requirements and maximizes power.

The parameter *α* in power prior method for controlling the degree of borrowing information is recommended to be 0.5. Considering that different *α* may have different effects on statistical performance, we conduct a sensitivity analysis on *α*. Results are summarized in Supplementary materials. We can see that the proposed design is robust to different *α* in terms of type I error, power, and sample size.

**Figure 3:**
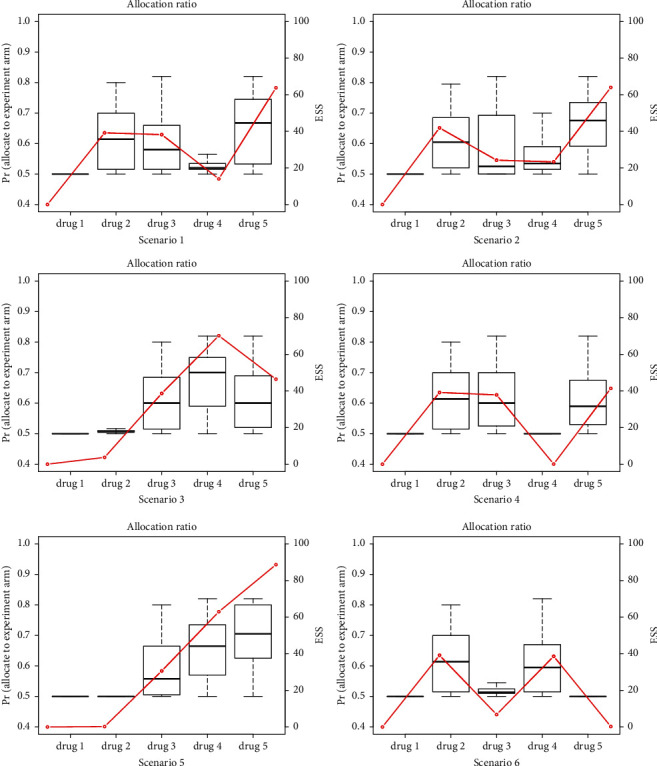
Proportion of patients assigned to the experimental arm and effective sample size using time trend calibration.

Furthermore, scenarios with baseline effectiveness drift are discussed. We run simulations for platform design using time trend calibration compared to noncalibration, in which *α* does not need to be prespecified. Results are shown in Table 2 and Figure 3. The time trend in the third column represents the baseline hazard of SOC. The underlined text represents the baseline hazard drifts. From the results of the Pr (reject *H*_0_) for two methods, we can see that the type I error is controlled at approximately 0.1 for time trend calibration when drift happens. While for the power prior, type I error is inflated due to the inconsistencies between stages. For example, in scenario 1, the parameter of time trend for drug 4 is 0.45, so SOC in drug 4 is more effective than that in others. Time trend calibration can identify such heterogeneity and choose to barely borrow information from previous stages, which can be confirmed in Figure 3.

We can see that drug 4's effective sample size and allocation ratio are both much lower than drugs 2 and 3. However, the power prior still borrows information, leading to the inflation of type I error. As for power, when the drug is truly effective, time trend calibration rejects the null hypothesis with a probability higher than 85%. The power prior may wrongly borrow information and lessen the effect size, resulting in lower power. Based on the results above, we can conclude that the time trend calibration is more robust to the baseline effectiveness drift. When drift exists between stages, time trend calibration is strongly recommended.

**Figure 4:**
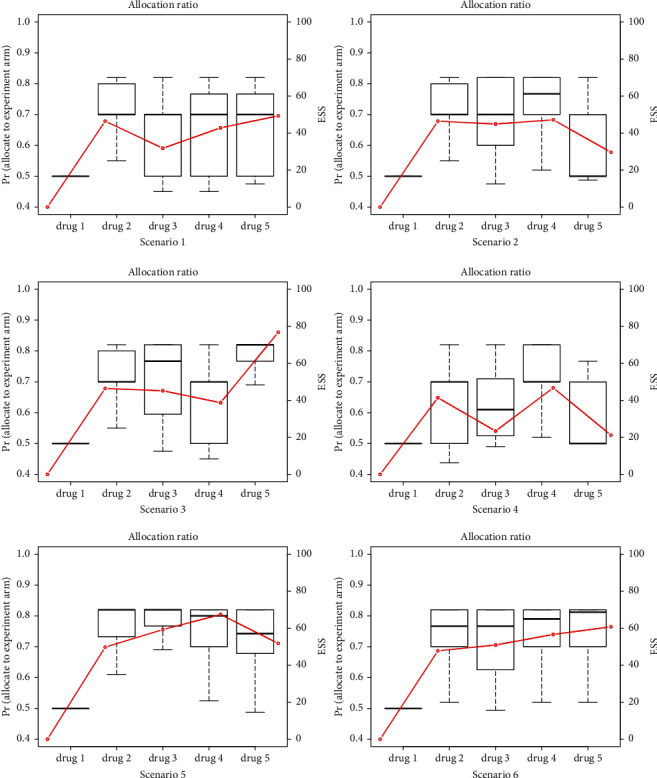
Proportion of patients assigned to the experimental arm and effective sample size in the optimal platform design.

The advantage of the proposed platform design is also reflected in the switch from effectiveness evaluation to optimal drug screening. The simulation results for the proposed platform design with optimal screening strategy are shown in Table 3 and Figure 4. We can see that in general, the optimal screening strategy has the highest probability to choose the most effective drug in different scenarios. Specifically, in scenario 1, when there is a relatively large effect size, the probability of selecting the most effective drug 2 can be as high as 93.9%. Different from the effectiveness evaluation procedure shown above, once selected as the optimal drug, it will be added into the control arm only with SOC. Therefore, the subsequent candidate drugs (drugs 3–5) in scenario 1 will be compared with drug 2 plus SOC, leading to a relatively high probability of early futility stopping. This shows an advantage of optimal screening design that drugs no better than the optimal drug will be excluded as soon as possible. Since the amount of borrowing information is a function of sample size in formula (10), arms with a high probability of early futility stopping have a smaller effective sample size.

Similarly, the upward trend of ESS in Figure 4 is not as obvious as that in Figures 2 and 3 because of the trade-off result between the smaller sample size and the accumulated amount of borrowed information. In other scenarios, with regard to different locations and sequences of optimal drug and different effect sizes, the proposed optimal drug screening procedure can always select the optimal drugs with the highest probability and early stop the trial for efficacy as long as there is enough evidence. The adaptive randomization rule can allocate more patients to the experimental arms, which is consistent with the previous simulation results.

## 4. Trial Illustration

**Figure 5:**
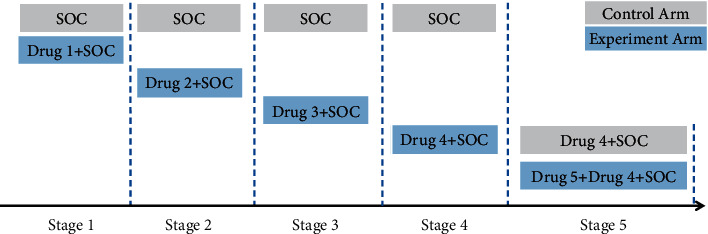
Diagram of COIVD-19 design for testing five drugs.

The famous COVID-19 drug candidates registered on clinicaltrial.gov are taken as an example to illustrate the proposed design with the comprehensive screening strategy. Suppose, the five drugs to be tested are Lopinavir, Favipiravir, CD24Fc, Remdesivir, and hydroxychloroquine. Their true clinical remission times are (5, 5, 5, 3.33, 5). Drug 4 Remdesivir can actually shorten the remission time and other drugs are ineffective. Then, the COVID-19 platform design is used to test 5 drugs sequentially. Results are shown in Figure 5.

The specific parameters in the test are listed in Table 4.

In the example, at the first stage, the probability that experimental arm A is more effective than the control arm B is 8.6%, so drug 1 is declared ineffective. Next stage, the control arm is still SOC, and the experimental arm is drug 2 + SOC. According to the posterior probability, that drug 2 is declared ineffective. The third stage is entered and drug 3 is found ineffective. In stage 4, the early stopping rule is triggered, so we end the fourth stage early and declare that drug 4 is effective. Drug 4 is added into the control arm and stage 5 is entered. At present, the control arm is drug 4 + SOC, while the experimental arm is drug 5 + drug 4 + SOC. The posterior probability of the experimental arm better than the control arm is 4.4%, so drug 5 is ineffective. The trial ends and we finally declare that of all 5 drugs, only drug 4 is effective. The total sample size of this trial is 874, which saves 126 patients compared with the traditional fixed design. From the example, we can see that the proposed design can stop early when the drug is sufficiently effective, speed up the trial process, save sample size, and meet ethical requirements.

## 5. Conclusions

Wave upon wave of COVID-19 outbreaks put heavy pressure on global disease burden and economics. Presently, there is nothing more important than controlling and ending the outbreak. Since no significantly efficacious treatment has been found yet, the development of new antivirus drugs is paramount to this end. In this situation, the traditional manner seems both time consuming and inefficient, so novel trial designs should be adopted to accelerate drug development. Therefore, we propose a platform design that evaluates multiple drug candidates in a unified design framework. Two drug development strategies are discussed here: the comprehensive screening strategy and the optimal screening strategy. The proposed design is able to tremendously shorten the overall trial duration and save the sample size for the control arm. Furthermore, the platform design incorporates an early stopping rule for significantly efficacious drugs, allowing patients to gain access to promising treatments as soon as possible, which helps control the spreading of disease. Simulation studies show that the design has good performance and robustness to different parameter settings. We adopt the power prior and time trend calibration to borrow information between different drugs, and more robust methods can also work well, such as commensurate prior [33] and robust meta-analytic-predictive prior [34], to further improve the performance of the design.

## Figures and Tables

**Table 1:** Operating characteristics of proposed platform design with comprehensive screening strategy.

				Proposed information-borrowing platform design						Nonborrowing
Scenario	Drug	Hazard ratio	Mean TTCR	Pr (reject*H*_0_)	Pr (early stopping for efficacy)	Pr (early stopping for futility)	*N*	*N*_*A*_	*N*_*B*_	Pr (reject*H*_0_)
1	1	1	5	0.076	0.035	0.107	186	93	93	0.086
	2	1.5	3.33	0.939	0.780	0.002	128	94	34	0.888
	3	1	5	0.048	0.017	0.113	186	134	52	0.084
	4	1	5	0.032	0.016	0.101	186	149	37	0.078
	5	1	5	0.043	0.017	0.086	188	152	36	0.068

2	1	1	5	0.087	0.029	0.098	186	93	93	0.092
	2	1	5	0.037	0.011	0.093	188	145	43	0.093
	3	1.75	2.86	0.999	0.987	0.000	81	57	24	0.984
	4	1	5	0.069	0.020	0.061	191	126	65	0.070
	5	1	5	0.031	0.008	0.061	191	153	38	0.078

3	1	1	5	0.081	0.032	0.114	186	93	93	0.077
	2	1	5	0.068	0.021	0.104	186	142	44	0.088
	3	1	5	0.050	0.020	0.107	185	148	37	0.087
	4	1.4	3.57	0.868	0.651	0.006	144	112	32	0.782
	5	1	5	0.054	0.020	0.116	185	137	48	0.064

4	1	1	5	0.079	0.031	0.111	186	93	93	0.067
	2	1.5	3.33	0.937	0.786	0.002	126	93	33	0.882
	3	1.5	3.33	0.929	0.754	0.000	132	93	39	0.901
	4	1	5	0.037	0.013	0.072	191	140	51	0.063
	5	1	5	0.026	0.009	0.076	191	154	37	0.047

5	1	1	5	0.081	0.035	0.098	186	93	93	0.082
	2	1.5	3.33	0.942	0.778	0.004	123	90	33	0.883
	3	0.8	6.25	0.005	0.005	0.585	144	100	44	0.006
	4	1	5	0.063	0.026	0.085	188	148	40	0.073
	5	1	5	0.046	0.024	0.095	186	150	36	0.082

6	1	1	5	0.081	0.035	0.098	186	93	93	0.077
	2	1.5	3.33	0.942	0.778	0.004	123	90	33	0.893
	3	0.8	6.25	0.005	0.005	0.585	144	100	44	0.003
	4	1	5	0.063	0.026	0.085	188	148	40	0.063
	5	1.5	3.33	0.962	0.832	0.002	122	93	29	0.889

*Note.* TTCR, time to clinical remission.

**Table 2:** Operating characteristics of time trend calibration compared with noncalibration.

					Time trend calibration						Noncalibration
Scenario	Drug	Time trend	Hazard ratio	Mean TTCR (T/C)	Pr (reject*H*_0_)	Pr (early stopping for efficacy)	Pr (early stopping for futility)	*N*	*N*_*A*_	*N*_*B*_	Pr (reject*H*_0_)
1	1	0.2	1	5/5	0.087	0.033	0.120	184	92	92	0.084
	2	0.2	1.5	3.3/5	0.951	0.811	0.002	127	79	48	0.922
	3	0.2	1	3.3/3.3	0.068	0.026	0.096	187	113	74	0.061
	4	0.45	1	1.48/1.48	0.119	0.097	0.007	190	102	88	0.961
	5	0.2	1	3.3/3.3	0.080	0.028	0.081	189	125	64	0.011

2	1	0.2	1	5/5	0.087	0.033	0.120	184	92	92	0.063
	2	0.2	1	5/5	0.072	0.019	0.106	187	115	72	0.053
	3	0.15	1.75	5.7/10	0.973	0.897	0.002	115	58	57	0.556
	4	0.2	1	2.85/2.85	0.095	0.045	0.081	186	96	90	0.621
	5	0.2	1	2.85/2.85	0.117	0.052	0.059	188	118	70	0.136

3	1	0.2	1	5/5	0.070	0.037	0.111	184	92	92	0.065
	2	0.45	1	2.2/2.2	0.063	0.036	0.051	190	97	93	0.910
	3	0.2	1	5/5	0.088	0.035	0.139	180	110	70	0.010
	4	0.2	1.4	3.57/5	0.888	0.715	0.003	139	94	45	0.427
	5	0.2	1	3.57/3.57	0.064	0.021	0.103	187	117	70	0.024

4	1	0.2	1	5/5	0.073	0.027	0.102	186	93	93	0.082
	2	0.2	1.5	3.33/5	0.948	0.834	0.001	123	77	46	0.943
	3	0.2	1.5	2.22/3.33	0.921	0.752	0.001	139	85	54	0.910
	4	0.089	1	5/5	0.073	0.027	0.109	186	93	93	0.004
	5	0.2	1	2.22/2.22	0.095	0.043	0.051	191	116	75	0.626

5	1	0.4	1	2.5/2.5	0.041	0.006	0.070	192	96	96	0.058
	2	0.2	1.5	3.33/5	0.904	0.699	0.004	142	71	71	0.427
	3	0.2	0.8	4.16/3.33	0.002	0.001	0.551	148	88	60	0.001
	4	0.2	1	3.33/3.33	0.081	0.028	0.104	186	123	63	0.018
	5	0.2	1	3.33/3.33	0.110	0.039	0.079	187	132	55	0.016

6	1	0.2	1	5/5	0.090	0.035	0.119	182	91	91	0.073
	2	0.2	1.5	3.33/5	0.953	0.822	0.001	124	77	47	0.940
	3	0.4	0.8	2.08/1.67	0.014	0.011	0.195	186	97	89	0.393
	4	0.2	1	3.33/3.33	0.079	0.023	0.130	184	111	73	0.011
	5	0.1	1.5	4.44/6.67	0.864	0.670	0.011	142	71	71	0.260

*Note.* TTCR (T/C), time to clinical remission of experimental arm and control arm, respectively.

**Table 3:** Operating characteristics of platform design with optimal screening strategy.

Scenario	Drug	Hazard ratio	Mean TTCR (T/C)	Prob (optimal)	Pr (early stopping for efficacy)	Pr (early stopping for futility)	*N*	*N*_*A*_	*N*_*B*_
1	1	1	5/5	0.013	0.032	0.105	186	93	93
	2	1.5	3.33/5	0.939	0.781	0.000	125	92	33
	3	1	5/3.33	0.000	0.000	0.916	93	64	29
	4	1	5/3.33	0.003	0.001	0.913	94	67	27
	5	1	5/3.33	0.045	0.002	0.923	90	64	26

2	1	1	5/5	0.000	0.032	0.105	186	93	93
	2	1.5	3.33/5	0.261	0.781	0.000	125	92	33
	3	2	2.5/3.33	0.735	0.455	0.005	162	115	47
	4	1.5	3.33/2.5	0.004	0.001	0.599	140	105	35
	5	1	5/2.5	0.000	0.000	0.999	62	40	22

3	1	1	5/5	0.000	0.032	0.105	186	93	93
	2	1.5	3.33/5	0.153	0.781	0.000	125	92	33
	3	1.7	2.94/3.33	0.219	0.133	0.022	185	133	52
	4	1	5/2.94	0.000	0.000	0.992	77	53	24
	5	2	2.5/2.94	0.628	0.360	0.005	173	138	35

4	1	1	5/5	0.000	0.032	0.105	186	93	93
	2	2	2.5/5	0.997	0.999	0.000	71	47	24
	3	1.7	2.94/2.5	0.003	0.001	0.379	164	105	59
	4	1.5	3.33/2.5	0.000	0.000	0.715	127	95	32
	5	1	5/2.5	0.000	0.000	1.000	56	34	22

5	1	1	5/5	0.000	0.032	0.105	186	93	93
	2	1.2	4.17/5	0.095	0.175	0.012	183	141	42
	3	1.3	3.85/4.17	0.227	0.219	0.021	178	139	39
	4	1.6	3.12/3.85	0.668	0.456	0.005	159	122	37
	5	1.2	4.17/3.12	0.010	0.001	0.636	136	101	35

6	1	1	5/5	0.000	0.032	0.105	186	93	93
	2	1.4	3.57/5	0.000	0.611	0.000	147	111	36
	3	1.8	2.78/3.57	0.024	0.428	0.006	162	118	44
	4	2.2	2.27/2.78	0.371	0.391	0.004	167	125	42
	5	2.7	1.85/2.27	0.605	0.349	0.007	173	132	41

**Table 4:** Parameters for decision between five drugs.

Metrics	Drug 1	Drug 2	Drug 3	Drug 4	Drug 5
Effective sample size	—	28.88	41.22	48.72	29.61
Sample size	186	190	187	124	187
Allocation ratio	0.5	0.72	0.78	0.72	0.68
Posterior probability	0.086	0.040	0.052	0.950	0.044
Decision	Ineffective	Ineffective	Ineffective	Effective	Ineffective

## Data Availability

The code used to support the findings of this study is available from the corresponding author upon request.

## Supplementary Materials

Supplementary Materials Supplementary tables and figures referenced in Section 3 are available.
