# Supplementary material for: An Adaptive Information Borrowing Platform Design for Testing Drug Candidates of COVID-19
Source: Can J Infect Dis Med Microbiol. 2022 Apr 22;2022:9293681. doi: 10.1155/2022/9293681 (PMC9029212; doi:10.1155/2022/9293681)
Supplement: Supplementary Materials — Supplementary tables and figures referenced in Section 3 are available. [file 9293681.f1.zip › 9293681.f1/Figure_supple2 (1).pdf]

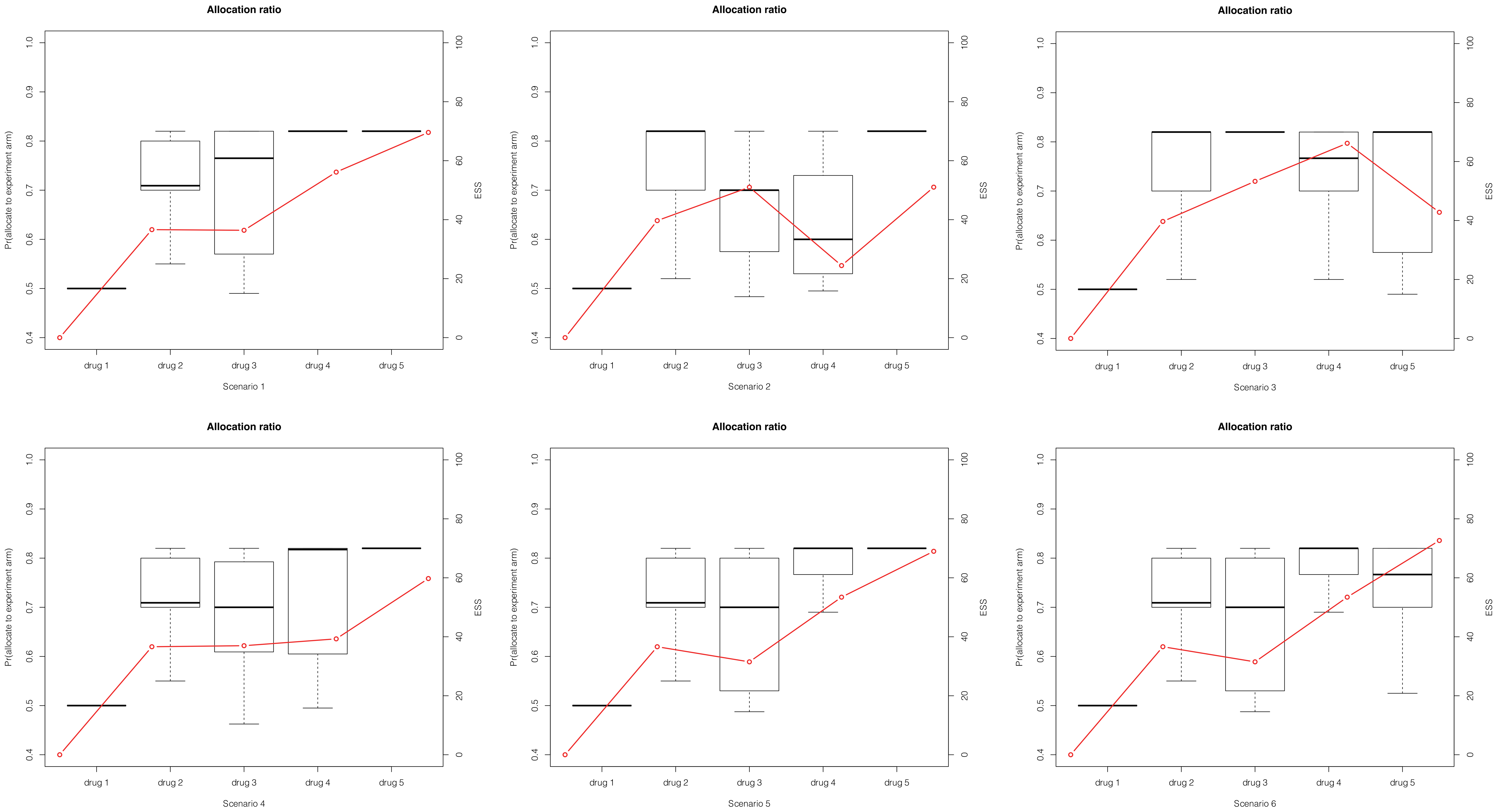

Allocation ratio
Pr(allocate to experiment arm)
ESS
1.0
0.9
0.8
0.7
0.6
0.5
0.4
100
80
60
40
20
0
drug 1
drug 2
drug 3
drug 4
drug 5
Scenario 1
Scenario 2
Scenario 3
Scenario 4
Scenario 5
Scenario 6
