# Supplementary material for: An Adaptive Information Borrowing Platform Design for Testing Drug Candidates of COVID-19
Source: Can J Infect Dis Med Microbiol. 2022 Apr 22;2022:9293681. doi: 10.1155/2022/9293681 (PMC9029212; doi:10.1155/2022/9293681)
Supplement: Supplementary Materials — Supplementary tables and figures referenced in Section 3 are available. [file 9293681.f1.zip › 9293681.f1/Supplementary file (for online publication only) (1).docx]

**Supplementary Data for “****An adaptive information borrowing platform design for testing drug candidates of COVID-19”**

We conduct additional sensitivity analysis to compare the statistical performance of the COVID-19 platform design at different when there’s no time trend calibration with comprehensive screening strategy. Results are tabulated below. We can see that our design is robust to different .

**Table S1 Operating characteristics of COVID-19 design when**

| Scenario | Drug | Hazard Ratio | Mean TTCR | Pr(reject ) | Pr(Early stopping  for efficacy) | Pr(Early stopping  for futility) |  |  |  |
| --- | --- | --- | --- | --- | --- | --- | --- | --- | --- |
| 1 | 1 | 1 | 5 | 0.066 | 0.027 | 0.105 | 186 | 93 | 93 |
|  | **2** | **1.5** | **3.33** | **0.908** | **0.724** | **0.001** | **134** | **98** | **36** |
|  | 3 | 1 | 5 | 0.057 | 0.019 | 0.086 | 188 | 131 | 57 |
|  | 4 | 1 | 5 | 0.039 | 0.012 | 0.076 | 189 | 149 | 40 |
|  | 5 | 1 | 5 | 0.046 | 0.021 | 0.081 | 187 | 151 | 36 |
| 2 | 1 | 1 | 5 | 0.066 | 0.027 | 0.105 | 186 | 93 | 93 |
|  | 2 | 1 | 5 | 0.057 | 0.022 | 0.079 | 189 | 137 | 52 |
|  | **3** | **1.75** | **2.86** | **0.999** | **0.973** | **0.001** | **87** | **62** | **25** |
|  | 4 | 1 | 5 | 0.060 | 0.020 | 0.060 | 192 | 118 | 74 |
|  | 5 | 1 | 5 | 0.038 | 0.015 | 0.058 | 191 | 150 | 41 |
| 3 | 1 | 1 | 5 | 0.092 | 0.030 | 0.095 | 188 | 94 | 94 |
|  | 2 | 1 | 5 | 0.055 | 0.021 | 0.090 | 187 | 135 | 52 |
|  | 3 | 1 | 5 | 0.043 | 0.014 | 0.079 | 189 | 149 | 40 |
|  | **4** | **1.4** | **3.57** | **0.846** | **0.593** | **0.005** | **147** | **115** | **32** |
|  | 5 | 1 | 5 | 0.056 | 0.020 | 0.110 | 185 | 134 | 51 |
| 4 | 1 | 1 | 5 | 0.086 | 0.037 | 0.114 | 186 | 93 | 93 |
|  | **2** | **1.5** | **3.33** | **0.911** | **0.710** | **0.001** | **135** | **98** | **37** |
|  | **3** | **1.5** | **3.33** | **0.926** | **0.723** | **0.001** | **136** | **93** | **43** |
|  | 4 | 1 | 5 | 0.039 | 0.006 | 0.069 | 191 | 132 | 59 |
|  | 5 | 1 | 5 | 0.035 | 0.007 | 0.069 | 192 | 153 | 39 |
| 5 | 1 | 1 | 5 | 0.089 | 0.036 | 0.111 | 186 | 93 | 93 |
|  | **2** | **1.5** | **3.33** | 0.902 | **0.734** | **0.000** | **133** | **97** | **36** |
|  | 3 | 0.8 | 6.25 | 0.004 | 0.004 | 0.552 | 147 | 100 | 47 |
|  | 4 | 1 | 5 | 0.038 | 0.013 | 0.065 | 191 | 148 | 43 |
|  | 5 | 1 | 5 | 0.045 | 0.018 | 0.067 | 190 | 153 | 37 |
| 6 | 1 | 1 | 5 | 0.086 | 0.030 | 0.109 | 186 | 93 | 93 |
|  | **2** | **1.5** | **3.33** | **0.898** | **0.697** | **0.000** | **138** | **101** | **37** |
|  | 3 | 0.8 | 6.25 | 0.001 | 0.002 | 0.578 | 144 | 99 | 45 |
|  | 4 | 1 | 5 | 0.043 | 0.013 | 0.088 | 189 | 147 | 42 |
|  | **5** | **1.5** | **3.33** | **0.956** | **0.816** | **0.002** | **125** | **95** | **30** |


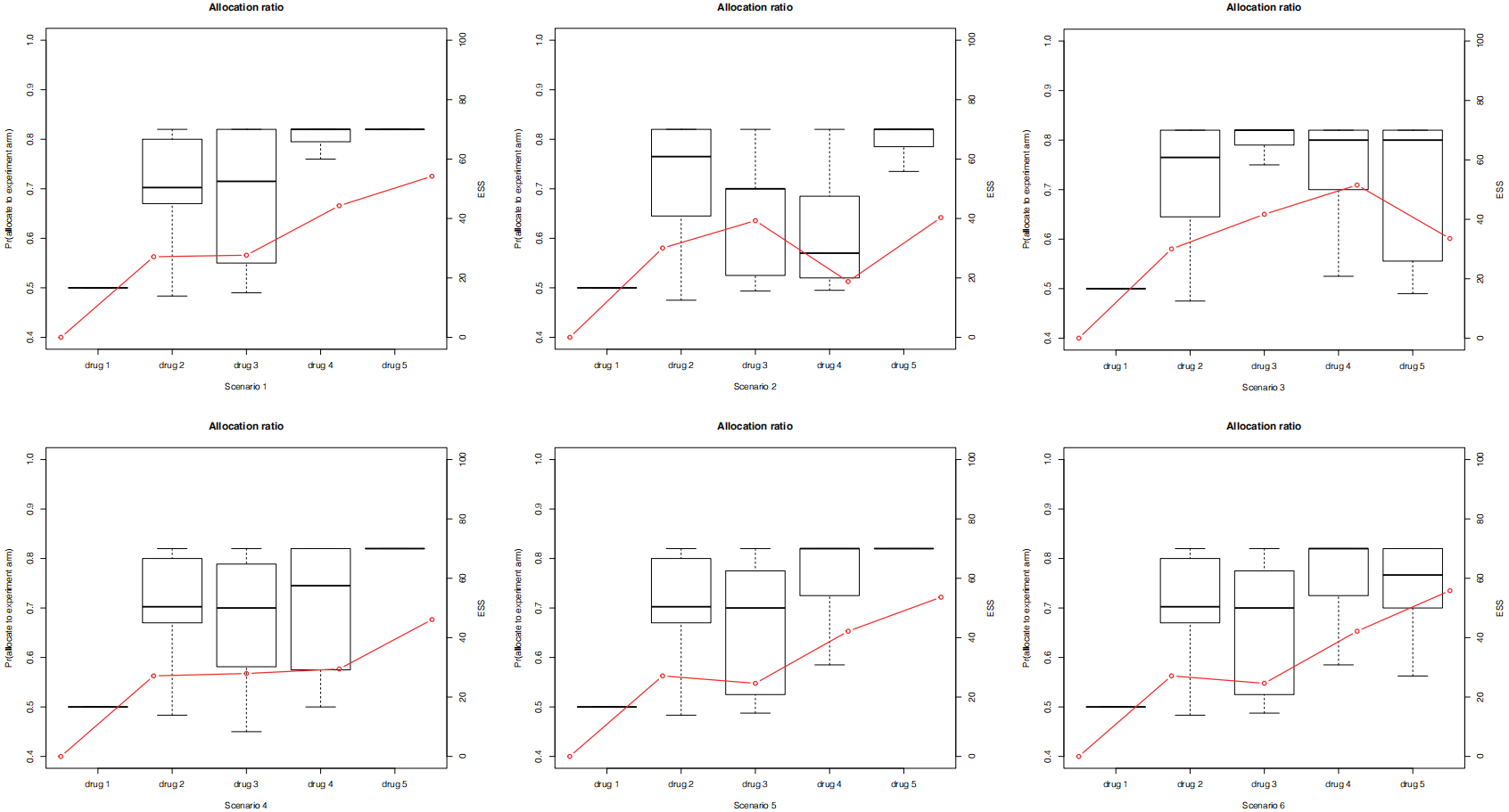


**Figure S1 Proportion of patients assigned to experiment arm and ESS when**

**Table S2 Operating characteristics of COVID-19 design when**

| Scenario | Drug | Hazard Ratio | Mean TTCR | Pr(reject ) | Pr(Early stopping  for efficacy) | Pr(Early stopping  for futility) |  |  |  |
| --- | --- | --- | --- | --- | --- | --- | --- | --- | --- |
| 1 | 1 | 1 | 5 | 0.088 | 0.036 | 0.097 | 186 | 93 | 93 |
|  | **2** | **1.5** | **3.33** | **0.928** | **0.757** | **0.002** | **129** | **95** | **34** |
|  | 3 | 1 | 5 | 0.041 | 0.013 | 0.094 | 188 | 134 | 54 |
|  | 4 | 1 | 5 | 0.035 | 0.014 | 0.086 | 188 | 150 | 38 |
|  | 5 | 1 | 5 | 0.025 | 0.010 | 0.086 | 189 | 153 | 36 |
| 2 | 1 | 1 | 5 | 0.079 | 0.034 | 0.101 | 186 | 93 | 93 |
|  | 2 | 1 | 5 | 0.053 | 0.023 | 0.099 | 186 | 140 | 46 |
|  | **3** | **1.75** | **2.86** | **0.998** | **0.969** | **0.000** | **86** | **61** | **25** |
|  | 4 | 1 | 5 | 0.073 | 0.019 | 0.060 | 192 | 123 | 69 |
|  | 5 | 1 | 5 | 0.048 | 0.012 | 0.078 | 189 | 150 | 39 |
| 3 | 1 | 1 | 5 | 0.078 | 0.035 | 0.104 | 184 | 92 | 92 |
|  | 2 | 1 | 5 | 0.042 | 0.017 | 0.105 | 186 | 140 | 46 |
|  | 3 | 1 | 5 | 0.042 | 0.016 | 0.092 | 187 | 149 | 38 |
|  | **4** | **1.4** | **3.57** | **0.859** | **0.635** | **0.002** | **144** | **113** | **31** |
|  | 5 | 1 | 5 | 0.038 | 0.010 | 0.099 | 189 | 140 | 49 |
| 4 | 1 | 1 | 5 | 0.076 | 0.036 | 0.119 | 184 | 92 | 92 |
|  | **2** | **1.5** | **3.33** | **0.927** | **0.762** | **0.002** | **130** | **95** | **35** |
|  | **3** | **1.5** | **3.33** | **0.916** | **0.722** | **0.001** | **140** | **98** | **42** |
|  | 4 | 1 | 5 | 0.033 | 0.008 | 0.078 | 191 | 139 | 52 |
|  | 5 | 1 | 5 | 0.023 | 0.003 | 0.073 | 192 | 155 | 37 |
| 5 | 1 | 1 | 5 | 0.077 | 0.036 | 0.105 | 186 | 93 | 93 |
|  | **2** | **1.5** | **3.33** | **0.914** | **0.736** | **0.001** | **132** | **97** | **35** |
|  | 3 | 0.8 | 6.25 | 0.002 | 0.002 | 0.581 | 141 | 98 | 43 |
|  | 4 | 1 | 5 | 0.045 | 0.020 | 0.064 | 190 | 149 | 41 |
|  | 5 | 1 | 5 | 0.038 | 0.011 | 0.072 | 191 | 154 | 37 |
| 6 | 1 | 1 | 5 | 0.073 | 0.038 | 0.107 | 184 | 92 | 92 |
|  | **2** | **1.5** | **3.33** | **0.919** | **0.758** | **0.000** | **130** | **96** | **34** |
|  | 3 | 0.8 | 6.25 | 0.000 | 0.000 | 0.595 | 141 | 99 | 42 |
|  | 4 | 1 | 5 | 0.046 | 0.019 | 0.079 | 188 | 147 | 41 |
|  | **5** | **1.5** | **3.33** | **0.950** | **0.795** | **0.000** | **132** | **102** | **30** |


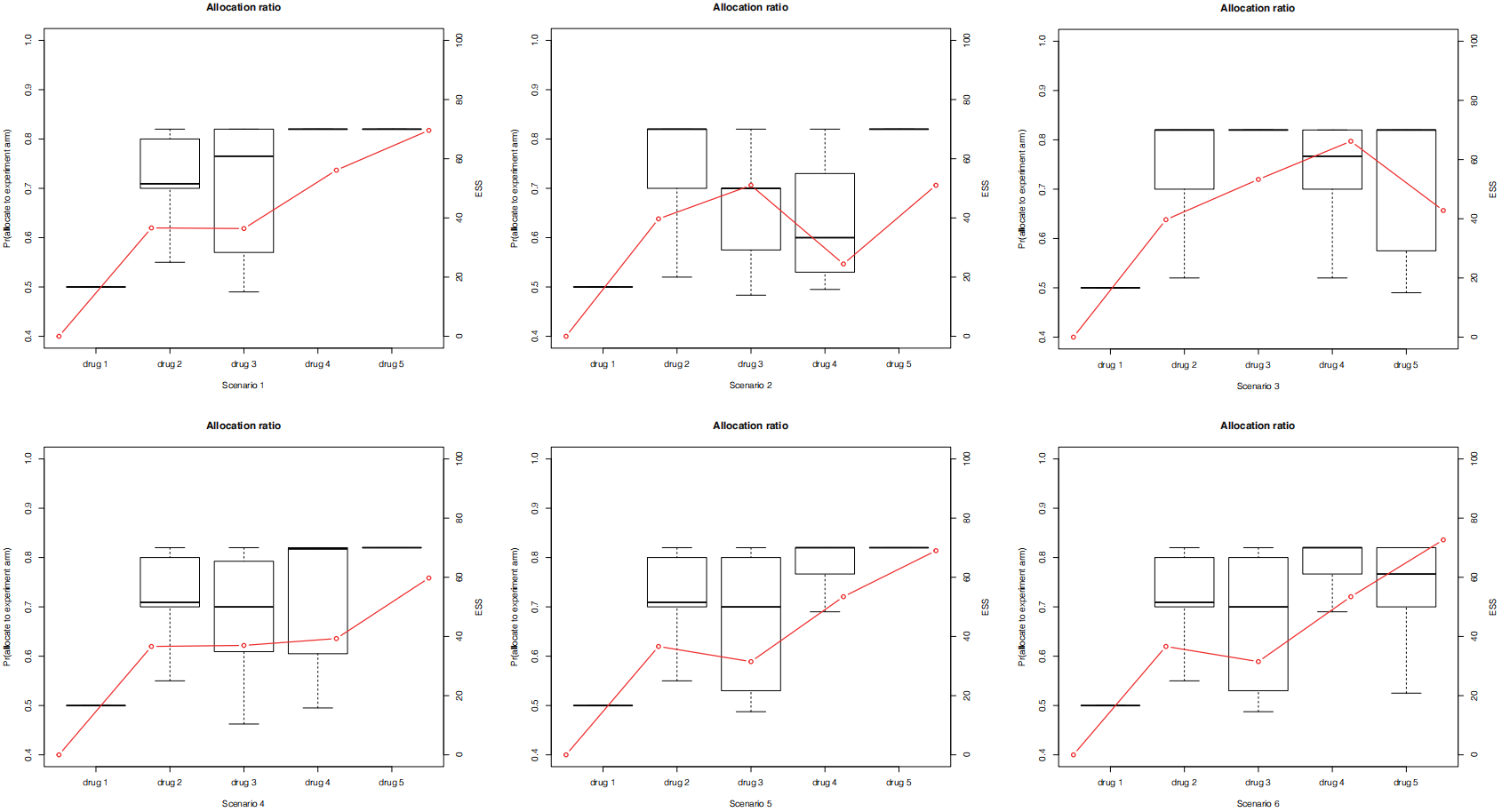


**Figure S2 Proportion of patients assigned to experiment arm and ESS when**

**Table S3 Operating characteristics of COVID-19 design when**

| Scenario | Drug | Hazard Ratio | Mean TTCR | Pr(reject ) | Pr(Early stopping  for efficacy) | Pr(Early stopping  for futility) |  |  |  |
| --- | --- | --- | --- | --- | --- | --- | --- | --- | --- |
| 1 | 1 | 1 | 5 | 0.082 | 0.030 | 0.101 | 186 | 93 | 93 |
|  | **2** | **1.5** | **3.33** | **0.934** | **0.762** | **0.002** | **128** | **95** | **33** |
|  | 3 | 1 | 5 | 0.051 | 0.013 | 0.097 | 188 | 138 | 50 |
|  | 4 | 1 | 5 | 0.049 | 0.012 | 0.112 | 186 | 150 | 36 |
|  | 5 | 1 | 5 | 0.031 | 0.012 | 0.114 | 186 | 151 | 35 |
| 2 | 1 | 1 | 5 | 0.074 | 0.030 | 0.103 | 186 | 93 | 93 |
|  | 2 | 1 | 5 | 0.056 | 0.022 | 0.119 | 184 | 143 | 41 |
|  | **3** | **1.75** | **2.86** | **0.997** | **0.982** | **0.000** | **84** | **59** | **25** |
|  | 4 | 1 | 5 | 0.053 | 0.011 | 0.076 | 191 | 131 | 60 |
|  | 5 | 1 | 5 | 0.040 | 0.007 | 0.089 | 189 | 152 | 37 |
| 3 | 1 | 1 | 5 | 0.091 | 0.030 | 0.104 | 186 | 93 | 93 |
|  | 2 | 1 | 5 | 0.048 | 0.022 | 0.103 | 187 | 145 | 42 |
|  | 3 | 1 | 5 | 0.035 | 0.016 | 0.108 | 186 | 150 | 36 |
|  | **4** | **1.4** | **3.57** | **0.882** | **0.694** | **0.003** | **140** | **109** | **31** |
|  | 5 | 1 | 5 | 0.035 | 0.013 | 0.101 | 188 | 141 | 47 |
| 4 | 1 | 1 | 5 | 0.077 | 0.029 | 0.101 | 186 | 93 | 93 |
|  | **2** | **1.5** | **3.33** | **0.944** | **0.790** | **0.001** | **123** | **91** | **32** |
|  | **3** | **1.5** | **3.33** | **0.915** | **0.751** | **0.001** | **128** | **98** | **40** |
|  | 4 | 1 | 5 | 0.034 | 0.005 | 0.088 | 190 | 144 | 46 |
|  | 5 | 1 | 5 | 0.028 | 0.004 | 0.077 | 191 | 155 | 36 |
| 5 | 1 | 1 | 5 | 0.083 | 0.033 | 0.101 | 186 | 93 | 93 |
|  | **2** | **1.5** | **3.33** | **0.942** | **0.793** | **0.002** | **127** | **94** | **33** |
|  | 3 | 0.8 | 6.25 | 0.002 | 0.002 | 0.623 | 138 | 99 | 39 |
|  | 4 | 1 | 5 | 0.035 | 0.017 | 0.106 | 186 | 148 | 38 |
|  | 5 | 1 | 5 | 0.049 | 0.024 | 0.102 | 186 | 150 | 36 |
| 6 | 1 | 1 | 5 | 0.075 | 0.032 | 0.103 | 186 | 93 | 93 |
|  | **2** | **1.5** | **3.33** | **0.928** | **0.798** | **0.001** | **125** | **92** | **33** |
|  | 3 | 0.8 | 6.25 | 0.001 | 0.001 | 0.626 | 139 | 99 | 40 |
|  | 4 | 1 | 5 | 0.040 | 0.016 | 0.099 | 188 | 149 | 39 |
|  | **5** | **1.5** | **3.33** | **0.952** | **0.822** | **0.001** | **124** | **95** | **29** |


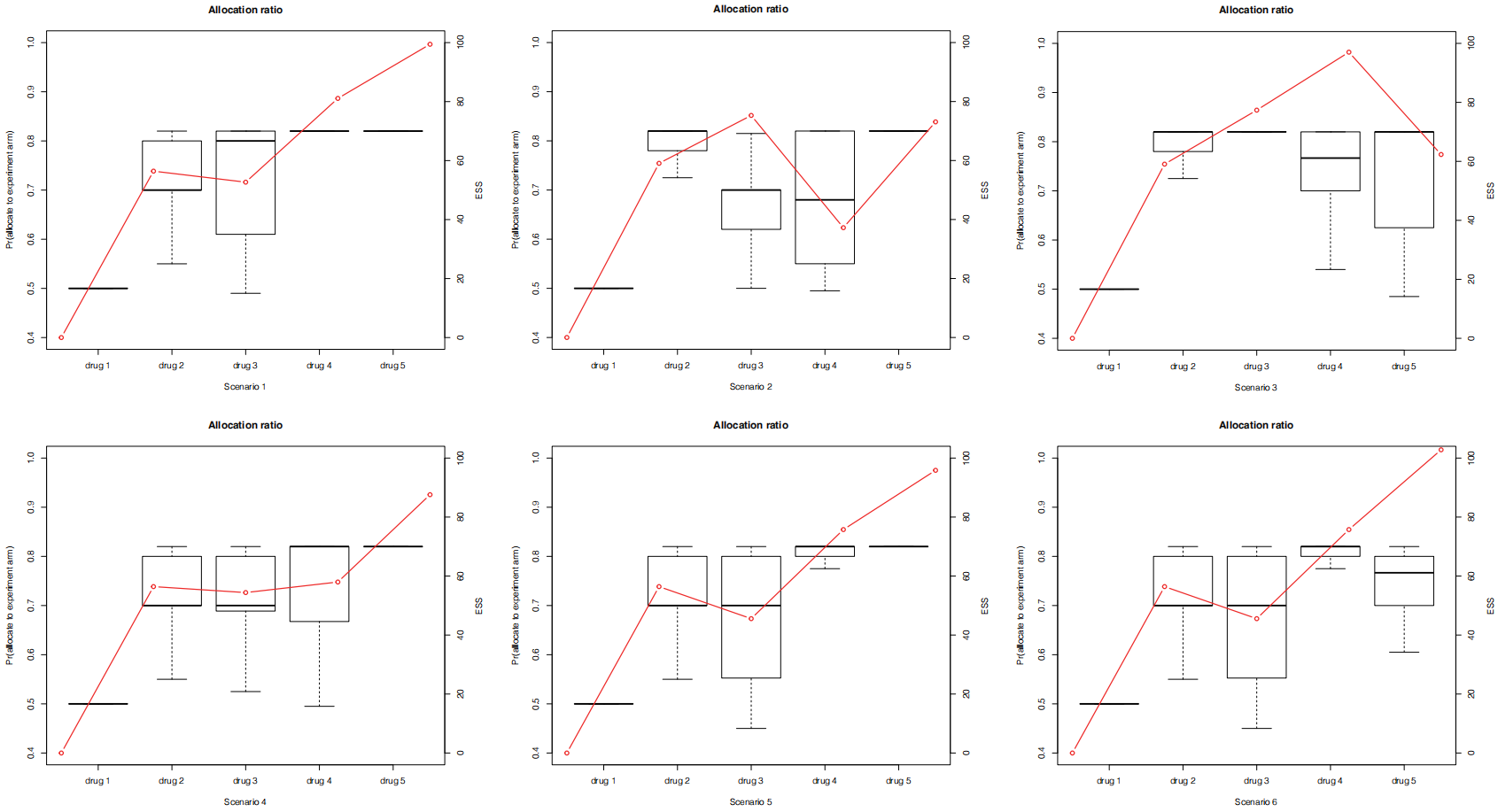


**Figure S3 Proportion of patients assigned to experiment arm and ESS when**
